# Judging Diatoms by Their Cover: Variability in Local Elasticity of *Lithodesmium undulatum* Undergoing Cell Division

**DOI:** 10.1371/journal.pone.0109089

**Published:** 2014-10-22

**Authors:** Lee Karp-Boss, Rachel Gueta, Itay Rousso

**Affiliations:** 1 School of Marine Sciences, University of Maine, Orono, Maine, United States of America; 2 Department of Structural Biology, Weizmann Institute of Science, Rehovot, Israel; 3 Department of Physiology and Cell Biology, Ben-Gurion University, Beer-Sheva, Israel; University of Ottawa, Canada

## Abstract

Unique features of diatoms are their intricate cell covers (frustules) made out of hydrated, amorphous silica. The frustule defines and maintains cell shape and protects cells against grazers and pathogens, yet it must allow for cell expansion during growth and division. Other siliceous structures have also evolved in some chain-forming species as means for holding neighboring cells together. Characterization and quantification of mechanical properties of these structures are crucial for the understanding of the relationship between form and function in diatoms, but thus far only a handful of studies have addressed this issue. We conducted micro-indentation experiments, using atomic force microscopy (AFM), to examine local variations in elastic (Young's) moduli of cells and linking structures in the marine, chain-forming diatom *Lithodesmium undulatum*. Using a fluorescent tracer that is incorporated into new cell wall components we tested the hypothesis that new siliceous structures differ in elastic modulus from their older counterparts. Results show that the local elastic modulus is a highly dynamic property. Elastic modulus of stained regions was significantly lower than that of unstained regions, suggesting that newly formed cell wall components are generally softer than the ones inherited from the parent cells. This study provides the first evidence of differentiation in *local* elastic properties in the course of the cell cycle. Hardening of newly formed regions may involve incorporation of additional, possibly organic, material but further studies are needed to elucidate the processes that regulate mechanical properties of the frustule during the cell cycle.

## Introduction

**Figure 1:**
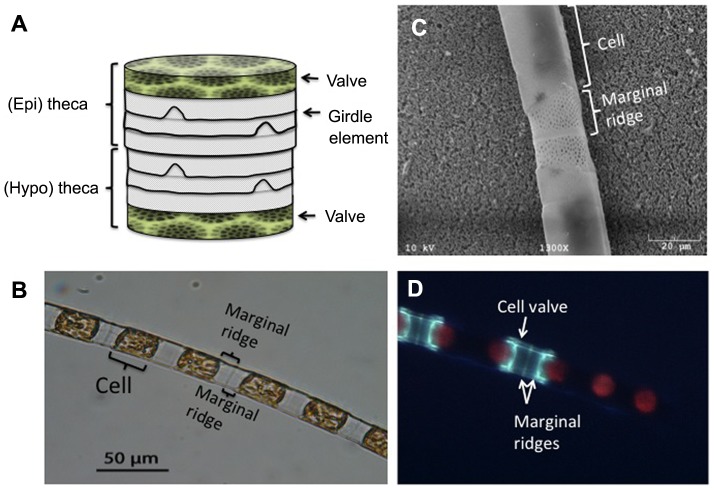
Frustule and chain morphology. (A) A schematic structure of the frustule (modified after [1]). The frustule consists of two overlapping elements, or thecae, that fit together like the two halves of a Petri dish. Each theca is capped with a distinctive structure called a valve. A series of thin, overlapping bands of silica, termed girdle elements (or girdle bands), extends from the rim of the valve forming the sidewall of the theca. The two thecae are identically shaped but differ slightly in diameter; one fits within the other, with their girdle elements partially overlapping, to form an enclosed structure around the cell's protoplasm. The frustule is perforated with nanometer-scale pores arranged in species-specific, ornate patterns that allow the exchange of solutes between the cell and the environment. (B) A light-microscope image of *L. undulatum*. (C) SEM image of *L. undulatum*. (D) Epifluorescence image of *L. undulatum* after staining with PDMPO. The green region indicates a valve and marginal ridges that were formed during the incubation time with PDMPO.

Diatoms have long been recognized as one of the most important groups of photosynthetic organisms in aquatic environments. They contribute significantly to primary production in oceans and lakes and consequently play significant roles in food-web dynamics and biogeochemical cycling. Diatoms' hallmarks are the intricate, silicified cell walls, or frustules, that they form (Figure 1). The frustule is a composite material, made primarily of amorphous, hydrated silica and organic compounds that are tightly associated with the silica [1]. The structure and biochemical composition of the organic matrix has not been fully characterized, but several organic compounds have been identified. These include highly modified peptides (silafins), long-chain polyamines and acidic polypeptides [1], [2], polysaccharides [3], [4], [5] and chitin [6].

Several hypotheses have been put forth on possible functions of the frustule [7]. Acting as a mechanical barrier and protecting the cell from grazers, viruses and parasites is the most apparent one. While field and laboratory observations show that the frustule does not provide complete protection against grazers and pathogens, it may still provide an ecological advantage by lowering such pressures [7], [8], [9]. It has also been suggested that the frustule provides a selective advantage by acting as ballast, removing planktonic cells from surface waters when growth conditions become adverse, and eliminating parasite-infected cells from surface populations [7]. Conversely, possessing a frustule sets some constraints on cell and population growth. Blooms of diatoms are restricted to times and locations where soluble silicon (silicic acid and silicate) is available. Healthy, growing cells need to compensate for the excess density of the frustule in order to remain suspended in the illuminated upper layers of oceans and lakes. Lastly, being encased in a rigid ‘glass house’ imposes restrictions on mechanisms associated with the cell cycle (division and growth). From an evolutionary perspective, structural and mechanical properties of the frustule must therefore reflect trade-offs between defense and growth. The structure of the frustule has been studied in great detail with light and electron microscopy [e.g., 10], and more recently with the use of atomic force microscopy [11], [12]. In contrast, mechanical properties of the frustule have been studied to a lesser extent, primarily due to the challenges associated with the application and detection of forces at scales relevant to the diatom cell. Improved knowledge of these properties is essential for better understanding the attributes of the frustule.

Two complementary approaches have been used to study mechanical properties of cells. In the first, the cell is considered as a whole and mechanical properties are studied integrally. For example, Hamm et al. [9] used calibrated glass micro-needles to determine the loads at which frustules of different species of diatoms break. They demonstrated that resistance to crushing by an applied force varies between species and with cell size [9], with larger cells crushing under lower force (90 µN) compared to smaller cells (750 µN). A second approach is to study local mechanical properties of the different elements that constitute the frustule. In the past decade, atomic force microscopy (AFM) has emerged as a promising tool for high-resolution studies of micro- and nano-mechanical properties of cells [13] and has been used to map hardness and elastic properties of frustules of several species of diatoms [14], [15], [16], [17]. Estimated values of the elastic modulus (for the few species that have been studied so far) vary by several orders of magnitude (Table 1). Differences in the degree of silicification between species [e.g., 16] and conditions under which measurements were taken (e.g., living cells in physiological media vs. fixed cells) have contributed to the large range of elastic moduli reported in the literature.

**Table 1:** A comparison between AFM estimated of the elastic modulus of diatoms.

Species	Young's modulus	Living or fixed cells	Cantilever-tip system	Reference
Navicula pelliculosa	7–100 s GPa	Cells treated with sulfuric acid to remove organic matrix, in air.	Conical tip, nominal radius 25 nm, spring constant 143 N/m.	[14]
*Coscinodiscus sp.*	1.5–15.6 GPa	Cells treated with sulfuric acid to remove organic matrix, in air.	Conical tip, nominal radius 25 nm. Spring constant 132 N/m.	[15]
*Phaeodactylum tricornutum*	0.25–0.75 MPa	Living cell in NaCl solution	Conical tip, nominal radius 20 nm, spring constant 0.01 N/m.	[16]
*Cylindrotheca closterium*	20–43 MPa (with rare soft spots: 2–5 MPa)100–150 MPa	Living cells in seawaterEmpty cells	Conical tip, nominal radius 8 nm, spring constant 5 N/m.	[17]
*Lithodesmium undulatum*	0.25–9 MPa	Living cells in seawater	2 µm spherical tip, average spring constant 4.5 N/m.	This study

Diatoms are unicellular protists, but colony formation is common. Several genera of diatoms evolved diverse siliceous and organic structures that extend from the frustule and link neighboring cells together to form elongated chains [10]. The use of AFM recently revealed a novel strategy in which loosely held cells are encased in an organic ‘sleeve’ [18]. Chain formation presents yet another dimension of complexity of biomechanics in diatoms. Chains suspended in the upper mixed layer of the ocean are subjected to hydrodynamic shearing forces, and their mechanical properties will ultimately determine their motion [19] and resistance to breakage by the flow [20]. Only recently has flexural stiffness (resistance to bending) of whole chains been quantified and shown to vary by more than 4 orders of magnitude between the examined species [20]. Global mechanical properties of chains integrate the properties of individual cells; each is constructed and assembled at nano-, meso- and micro-scales [1]. Understanding the functional morphology of chains requires characterization of mechanical properties at multiple scales.

Here we conducted AFM micro-indentation experiments to study elastic moduli of cells and their linking structures in the marine, chain-forming species *Lithodesmium undulatum* (Figure 1), and examined whether cell division results in variations in local elasticity. When a diatom cell divides, each daughter cell inherits one valve from the parent cell and forms a new valve and linking structures. New girdle elements are added through the cell cycle or at particular stages, depending on the species [10]. Thus, structural constituents of a chain differ in ‘age’ and potentially in elastic properties. To test this hypothesis we labeled newly formed regions of the frustule and marginal ridges with a fluorescent dye and compared their elastic moduli to those of older regions.

## Methods

### Species and sample preparation

*L. undulatum* is a chain-forming, marine planktonic species, found in tropical and temperate waters [21]. This species was chosen because of the relatively large size of its cells and linking structures between cells, which makes it easier to target specific structural features. Flexural stiffness of whole chains has been estimated for this species in a previous study [20]. *L. undulatum* cells are rectangular or square in girdle view and triangular (sometimes quadrangular) in valve view. A porous, siliceous ridge (called marginal ridge) surrounds the edges of the valve, extending toward the neighboring cell and merging with it to form rigid chains (Figure 1).

Non-axenic cultures of *L. undulatum* (CCMP1806) were grown in sterile L1 media ([22]; nutrients stocks were obtained from the National Center for Marine Algae and Microbiota, Bigelow Laboratory for Ocean Sciences, Maine, USA) made from filtered seawater (collected from the Gulf of Maine). The same source (carboy) of seawater was used for preparing all the media used in this study to ensure that salinity was the same in all stock cultures. Cultures were kept at 20°C under illumination of 80–100 µmol photons m^−2^s^−1^. Cells were maintained in exponential growth by diluting stock cultures with fresh media, every 1–2 d.

Central to obtaining successful AFM measurements is that cells or chains are firmly attached to a substrate (e.g., Petri dish, microscope slide). Several adhesives, commonly used in AFM experiments (polylysin, biobond, mica, HMDS and BD Cell-Tak), were tested in preliminary experiments. The best adhesion was obtained with BD Cell-Tak, using the following procedure: 10 ml aliquots were drawn from an exponentially growing culture and centrifuged at 1000 rpm for 5 min. Seven milliliters of supernatant were carefully removed from the centrifuge tube and replaced by 7 ml of DIW. The diatom pellet at the bottom of the tube was gently, but thoroughly, mixed and the sample was centrifuged again at 1000 rpm for 5 min. To concentrate chains, 9 ml of supernatant were carefully removed and the diatom pellet at the bottom of the tube was gently mixed to obtain a 1 ml, concentrated sample of chains. An area of ∼1.5×1.5 cm in the center of a Petri dish was coated with BD Cell-Tak and the sample was immediately placed on top of the coated area. Cells were allowed to settle and adhere to the Petri dish for 3 min. Lowering salinity of the media at the time chains initially come in contact with Cell-Tak appears to be essential for strong adhesion. To remove unattached or weakly adhered cells the Petri dish was rinsed 3 times with fresh, L1 media (from the same stock as the growth media) and then filled with L1 media. Visual examination under a microscope revealed no broken cells, but the cell protoplasm contracted into a tight ball, suggesting that cells were stressed. To restrict the stress, this procedure was done as quickly as possible; cells were exposed to reduced salinity for approximately 10 min. Upon return into L1 media the protoplasm began to expand, and cells regained their native look within 30–60 min. It is likely that long chains broke during centrifugation but chains with up to 10 cells remained abundant in the sample.

### Cell staining with PDMPO

The fluorophore PDMPO [2-(4-pyridyl)-5([4-dimethylaminoethyl-aminocarbamoyl)-methoxy]phenyl) oxazole], also named LysoSensor Yellow/Blue DND–160, has been successfully used as a tracer of silica deposition in diatoms [23], [24], [25]. PDMPO enters cells quickly, accumulates in the silica deposition vesicle (SDV) and gets trapped in the new frustule components that are being formed in the SDV. The probe was obtained from Molecular Probe, OR (L-7545) and was used according to manufacturer recommendations, at a final concentration of 0.25 µM. This concentration was found to produce consistent and long-lasting staining, without any deleterious effects on cells and their growth [24]. Cells were incubated with the probe for 4 h prior to measurements. Excess stain was rinsed when samples were prepared for the indentation experiments, as described above.

### AFM indentation experiments

All measurements were conducted on living cells immersed in liquid (L1 media). The experimental system consisted of a Bioscope atomic force microscope, with a Nanoscope IV controller (Veeco, Santa Barbra, CA) and a dimension XY closed-loop scanner, mounted on a motorized inverted microscope with phase/epifluorescence imaging (Axiovert 200M, Carl Zeiss, Heidelberg, Germany). Indentation experiments were carried out with silicon nitride cantilevers with a borosilicate spherical tip (radius: 2 µm; Novascan Technologies, Inc. IA, USA). Spring constants of cantilevers were determined experimentally by measuring their thermal fluctuations [26]. Spring constants ranged from 4.3 (±0.2) to 4.8 (±0.1) N/m. The AFM was operated in force-distance mode. With the aid of the optical microscope on which the AFM was mounted, chains were positioned under the edge of the cantilever, and the cantilever was carefully lowered until the tip came into contact with the sample. Locations of newly forms structural components were determined by switching the microscope into epifluorescence mode. The location of the tip on the cell/chain was verified visually. At each location approximately 100 successive force-distance curves were collected at a scan rate of 2 Hz. A clean glass slide served as a hard reference sample; a drop of media was placed onto the slide, and 10–20 successive force-distance curves were collected at three different locations on the slide, at the beginning and the end of each set of measurements. To maintain a constant range of loading forces the maximal deflection of the probe was limited by a trigger of 0.5–0.6 V, resulting in maximal loading forces that ranged between 200–400 nN. The applied forces are 3 orders of magnitude smaller than loading forces that were required to crush frustules of two other species of diatoms (90–700 µN; [9]). For each chain, force-distance curves were collected along the chain, sampling the following structural components: the girdle (at the center of the cell), the girdle-valve mantle transition zone, the valve mantle-marginal ridge transition zone (at the valve edge), and the marginal ridge. In preliminary experiments (data not shown) we conducted high-resolution mapping of the stiffness of specific regions of the frustule (e.g., girdle element, marginal ridge; cultures were not incubated with PDMPO) and found that local stiffness within a cell varied by a factor of 4 while variation in local stiffness between cells was higher (a factor of 10). High-resolution mapping is time consuming, and for the question addressed here samples from a large number of cells were required. To restrict the time that cells were under stress and to increase statistical power (to sample larger numbers of different cells) the number of samples per cell was reduced to one per location, and data from different cells were pooled to test for regional differences. Because chains always settled on their girdle side it was not possible to conduct measurements on the valve face.

### Data analysis

**Figure 2:**
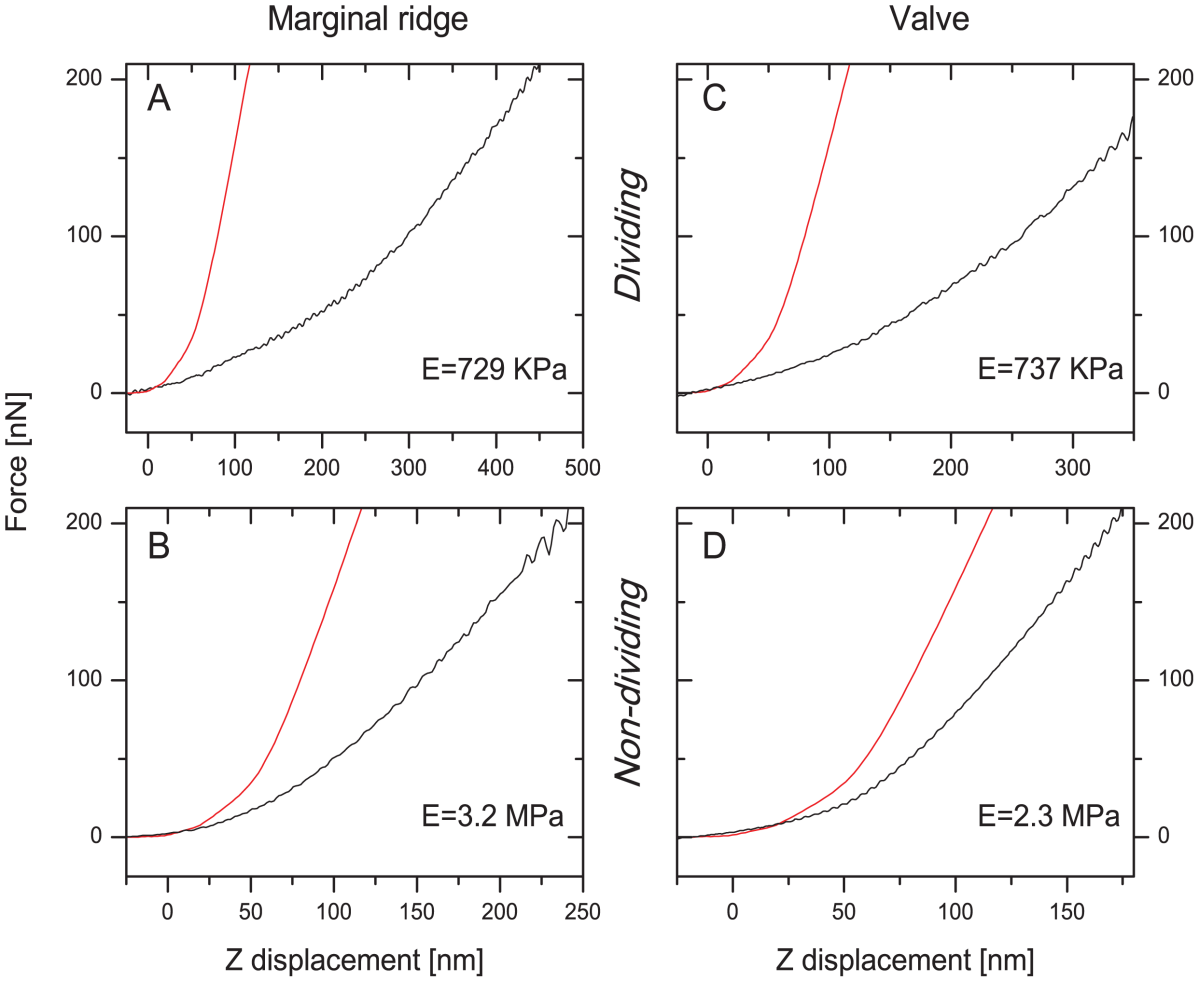
Measuring the stiffness of the cell using indentation type experiments. Averaged force distance curves for deflection of the cantilever (reference on a glass slide; red curve) and for a cell (black curve) measured in the marginal ridge (A) and valve (B) regions. To set the tip-sample contact point (Z_0_) to a distance of zero, the curves were shifted along the Z-axis. Approximately 100 consecutive curves were acquired for each experiment. The indentation depth is defined as the difference between the Z position of the cell and cantilever deflection at a given loading force. The Young's modulus of the sample was determined by fitting the data with the Hertz model (see Methods).

Prior to analysis, each set of successive curves was carefully reviewed and examined for shifts in behavior that might indicate potential damage to the sampled area in the course of the measurement. Sets for which shifts in the shape of the curve were detected and/or the spread between curves was large were discarded from the analysis. Only the approaching part of the force-distance curves was used for analysis. Each curve from a set of successive force-distance curves was shifted to set the deflection in the non-contact mode to zero. Averaged force-distance curves were then converted from deflection units (V) to loading forces (N) by multiplying by the deflection sensitivity (in nm/V, derived from a force-distance curve performed on glass) and the spring constant of the cantilever (N/m). Example of force-distance curves from valve and marginal ridge regions are presented in Figure 2. Young's moduli (elastic moduli, E) were estimated by fitting a modified Hertz model, for a spherical probe [27], to the averaged force-distance curves, using a custom MATLAB code. Samples that did not show good fit to the model were discarded because their E could not be estimated. E values for the different structural features, as well as stained and non-stained regions were grouped. Statistical differences between means and medians, respectively, were tested using parametric (ANOVA) and nonparametric (Kruskal-Wallis) statistics at a level of ≤0.05.

## Results and Discussion

**Figure 3:**
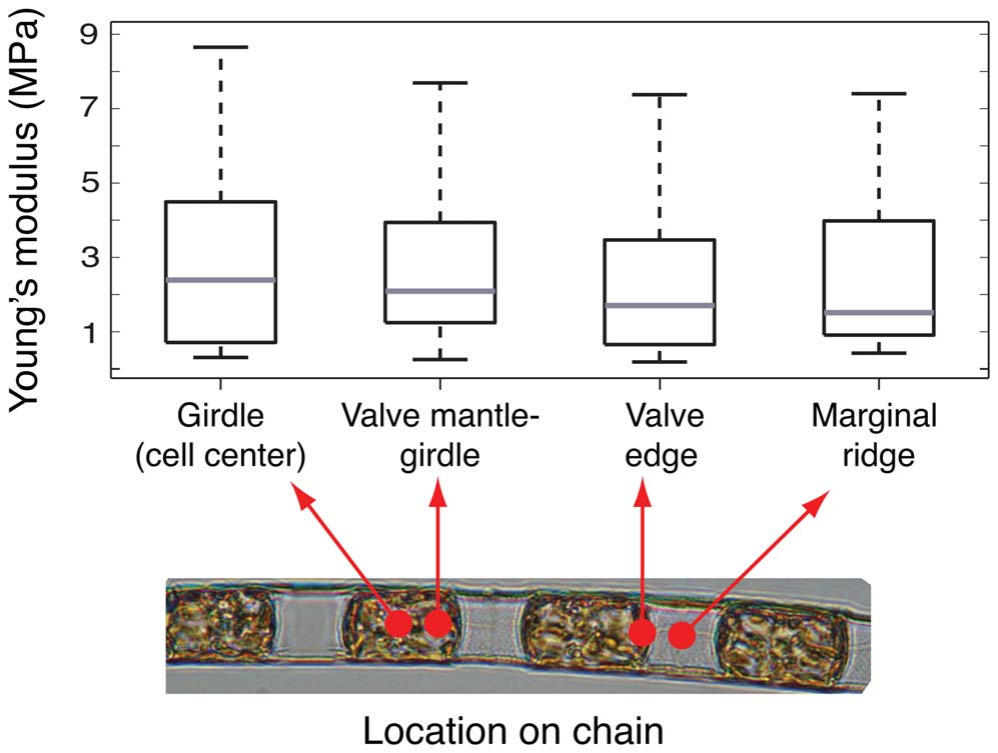
A box-and-whisker plot of the Young's modulus (E) as a function of location on the chains (girdle n = 15 samples; valve mantle-girdle transition n = 31 samples; valve edge n = 21 samples; marginal ridge n = 25 samples). On each box, the central gray line marks the median value, the edges of the box are the 25th and 75th percentiles, and the whiskers extend to the most extreme data points the algorithm considers not to be outliers.

**Figure 4:**
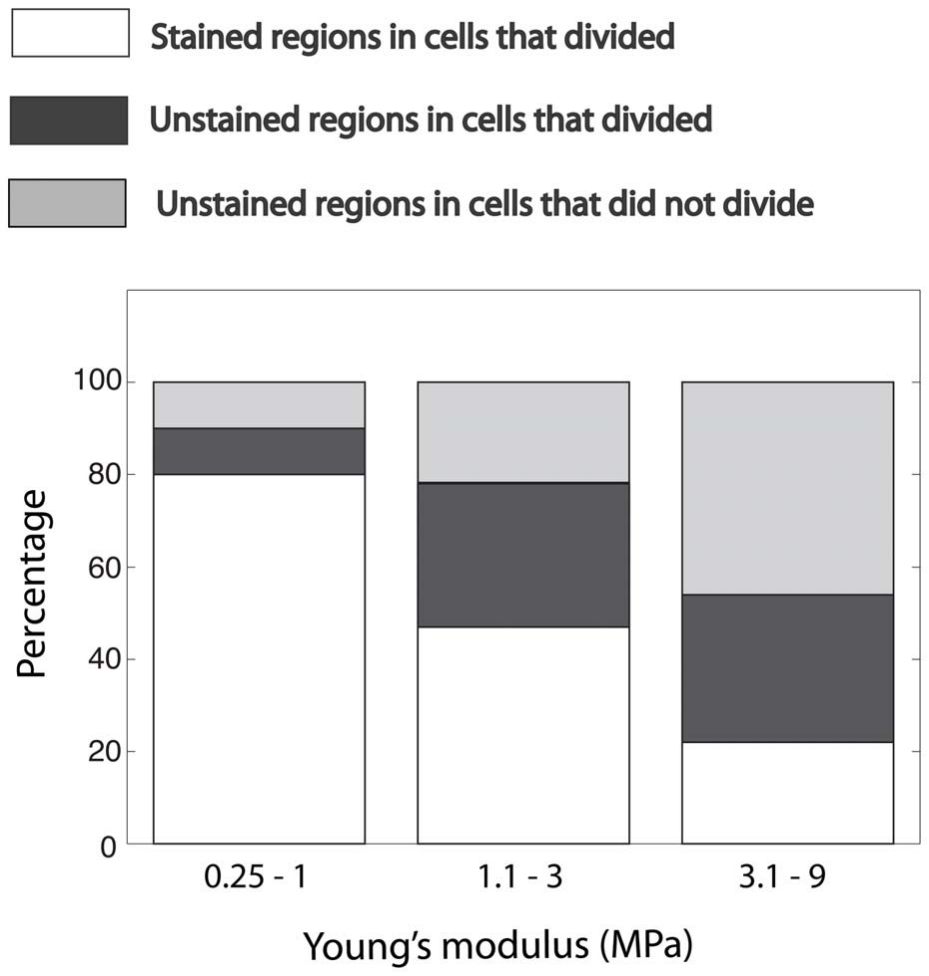
Frequency distribution of Young's moduli (E) for stained and unstained structural components. Data were grouped into 3 ‘stiffness’ categories. Number of samples in each category: 0.3<E<0.99 MPa n = 20; 1<E<2.99 MPa n = 32; E>3 MPa n = 37.

Measurements were conducted on short chains of *L. undulatum* (2–6 cells per chain). Overall, local elasticity, parameterized as Young's modulus (E), was highly variable, spanning a wide range from 0.25 to 9 MPa (n = 85 locations; 35 cells from 14 chains were analyzed in total). Within a single chain, local stiffness varied by a factor of 3–6. When grouped according to location (girdle at the cell center, valve mantle, valve edge and marginal ridge) there was no significant difference in mean Young's modulus between locations, but variability within each region was high (Figure 3). However, when the elasticity of stained vs. unstained regions was compared, patterns began to emerge. Young's modulus values from all sampling locations were grouped into 3 ‘stiffness’ categories: E≤1 MPa (‘soft’), 1.1<E≤3 MPa (‘intermediate’) and 3<E≤9 MPa (‘stiff’). Within the ‘soft’ group, 80% of the samples were associated with stained regions while the ‘stiff’ group primarily comprised samples from unstained regions (78%, Figure 4). Overall, Young's modulus of stained regions was significantly lower than that of unstained regions (Kruskal Wallis P<0.01, ANOVA P<0.01). For cells that divided during the period of incubation, newly formed thecae and marginal ridges were significantly ‘softer’ than their counterparts inherited from the parent cell (Kruskal Wallis P = 0.03; ANOVA P = 0.02). Values from the latter were not significantly different from values obtained from cells that did not divide (Kruskal Wallis/ANOVA P = 0.25).

A handful of studies have used AFM nanoindentation experiments to characterize the elastic moduli of different species of diatoms (Table 1). Because experimental conditions vary among these studies (e.g., growth conditions, choice of probe and AFM settings, sample preparation, design of measurements, etc.) comparisons of Young's modulus can be only relative. Estimates of Young's modulus for *L. undulatum* are intermediate between values reported in two other studies that examined living diatom cells, and several orders of magnitude lower than values reported for cells that were treated with acid to remove the organic matrix (Table 1). Young's modulus reported for cells treated with acids are consistently higher than those reported for living cells, presumably due to the absence of cytoplasm and structural modifications to the frustule caused by the removal of the organic matrix [17].

In this study we found no significant difference between mean Young's modulus of frustule constituents and marginal ridges in chains (Figure 3). This result is somewhat surprising, as one would expect regions of distinct morphology and function to also differ in their elastic properties. Previous studies have reported spatial variability in the elastic modulus in association with structural features [14], [16], [17]. For example, the girdle region of the single-celled, pennate diatom *Phaodactylum tricornutum* was found to be significantly softer than the valve, presumably due to differences in the degree of silicification between these two [16]. However, in other cases (*Cylindrotheca closterium*) average Young's modulus of the valve and girdle region were not significantly different [17]. Our observed difference in elastic moduli of stained vs. unstained regions of chains suggests that micromechanical properties are not fixed features of specific structures but rather properties that dynamically change with the cell cycle.

A concern is that the incorporation of PDMPO into new structural components could have altered their mechanical properties. In our preliminary experiments (see Methods) chains were not incubated with PDMPO prior to measurements yet the range of Young's modulus values obtained in these experiments (0.3–5MP) was similar to the one reported here (Figure 4). If PDMPO had a significant effect on the mechanical properties of newly formed structures (stained regions), making them ‘softer’ than unstained regions, then we would expect to see a narrower range of values in our preliminary experiments. Low Young's modulus values obtained in preliminary experiments likely represent newly formed sections but we could not confirm that because cultures were not incubated with the stain.

Abiotic factors such as salinity and availability of iron in the growth media where shown to affect the nanostructure and mechanical properties of the frustule [28], [29]. All cells used in this study were kept under the same growth conditions throughout the duration of experiments. Cells were briefly exposed to low salinity during sample preparation (see Methods). If a short-term (less than 10 minutes) change in salinity has any effect on the properties of the silica or the organic material associated with it we would expect to see a similar effect on all cells. Therefore, it is not likely that the differences we observed between newly formed structural components and their older counterparts are due to abiotic factors such as short-term changes in salinity.

### Elastic modulus and the cell cycle

Some of the processes associated with the diatom cell cycle are inherently mechanical and as such they must entail mechanical regulation of the cell wall. Following mitosis and cleavage each daughter cell begins to form its new valve while still enclosed within the parental cell wall. Morphogenesis of the valve is carried on within a specialized, membrane-bound organelle, the silica deposition vesicle (SDV), a process that is tightly controlled by actin and microtubule networks [30], [31]. When fully formed, or slightly before maturity, the SDV membrane fuses with the cell membrane, and the new valve is released. Girdle bands are being added after daughter cells have been separated and in the process the two thecae slide apart to allow for cell expansion along the axis of division. The entire cell cycle ranges from less than one to a few days, varying with growth conditions and species. The duration of the morphogenesis of specific structures (e.g., valve, girdle and linking structures in chains) has been documented for only a few species. In the pennate diatom *Navicula salinarum* valve morphogenesis, from the initial time of incubation with a the fluorescence tracer PDMPO until the two new valves begin to separate, lasted for about 4 h [25]. A shorter period was estimated for valve formation in the centric diatom *Thalassiosira pseudonana* (<30 min); rate of girdle band formation in this species was estimated to be on the order of 1 band per 30 min [32].

In this study, cells were incubated with PDMPO for 4 h and AFM sample preparation, and measurements commenced at the end of the incubation period. Growth in cultures was not synchronized thus stained and unstained cells represent different stages in the cell cycle. Here we show, for the first time, that local elasticity continuously changes during the cell cycle. The processes that are involved and the mechanisms that control these changes are yet to be discovered, but likely to entail the remodeling of cell wall components outside the SDV. Young's moduli in the ‘soft’ category, that encompassed the majority of the newly formed (stained) structures, ranged between 0.25 and 0.9 MPa. These values are of the same order of magnitude as values reported for the poorly silicified diatom *P. tricornum* [16]. Because deposition of silica is thought to take place only within the SDV [3], we do not think that in the case of *L. undulatum* newly released valves, girdle elements or marginal ridges are initially weakly silicified and later harden due to further deposition of silica, though we cannot completely rule out this possibility without direct chemical analysis. Alternatively, the observed changes in local elasticity could result from structural modifications to organic components of the cell wall. Processes involved may include the formation of transient layers via differential regulation of genes that encode the synthesis and hydrolysis of different organic polymers (e.g., proteins, polysaccharides and chitin). Current understanding of the formation and dynamics of the organic matrix remains rudimentary but recent observations are in support of this hypothesis. Tesson and Hildebrand [5] documented the presence of transient polysaccharide structures and suggested that different layers might be deposited at different times during the formation of the cell wall. Not all of the organic components that they identified were directly associated with silica morphogenesis and some appeared to have only a structural role [5]. Proteins, other than those associated with silica morphogenesis in the SDV, may also play a role in regulating mechanical properties locally. Pleuralins, for example, are not incorporated into the girdle via the SDV pathway but rather they are secreted in the region of the cleavage furrow during cell division. Their localization in association with terminal girdle bands of the two thecae varies during the cell cycle [33]. It is possible that in some cases stained regions were still enclosed with the structure of the parent cell and what we sampled is a segment of the old terminal girdle band. In that case, alteration of cell wall proteins, like pleuralins, and/or local degradation of the organic matrix (e.g., by specific proteases) may permit local relaxation of the rigid structure during sliding and separation of the thecae. Whole transcriptome analysis during a synchronized cell-cycle progression in the diatom *T. pseudonana* recently alluded to the potentially significant role of protein degradation in re-organization of cell wall components [34]. Remodeling of structural components of the cell wall is likely to entail those organic compounds and processes that are involved in higher-order structural formation; proteins and polysaccharides that are directly involved in the nano-scale polymerization of the silica within the SDV may become incorporated in the organic matrix without additional modifications. A critical step for obtaining further insights into how the architecture and mechanical properties of the frustule might be modified during the cell cycle will be to identify enzyme complexes and processes that regulate the synthesis and hydrolysis of cell wall polymers.

## Conclusions and Future Prospects

Here we show that the local elastic modulus is a highly dynamic property, and provide the first evidence of differentiation in *local* elastic properties in the course of the cell cycle. Furthermore, results from this study suggest that micromechanical properties of chain-forming species are neither uniform, nor related to specific structural constituents of the chain. Rather, the stiffness of segments of chains changes dynamically as new units are added. Local variations in elastic modulus observed in this study are larger than the factor-of-two variations in flextural stiffness reported for whole chains of *L. undulatum* [20]. If in an exponentially growing culture of chains a similar proportion of new and old units in a chain is maintained, then integrated mechanical property of whole chains (e.g., their flexural stiffness) would not show large variations.

This study is a first step in characterizing the local mechanical properties of chains and their relation to the cell cycle. Clearly, additional studies with different species are needed to examine how general are the patterns observed here and evaluate implications to ecological processes such as interactions with ambient flows and predators. Elucidating the interplay between material properties and the cell cycle will require integration of structural, mechanical and molecular studies. Molecular-level understanding of frustule morphogenesis has advanced significantly in the past decade, including the characterization of cell wall proteins [1], [2] and polysaccharides [5], and whole transcriptome analysis during silica starvation and recovery [34]. Application of genomic and proteomic approaches to better understand the regulation of different proteins that are associated with the cell wall, during the entire cell cycle, in conjunction with measurements of mechanical properties could prove particularly valuable.
